# Serum immunoglobulin concentrations and risk of type 2 diabetes mellitus in adults: a prospective cohort study from the TCLSIH study

**DOI:** 10.1186/s12865-024-00637-9

**Published:** 2024-07-29

**Authors:** Li Zhang, Yuanbin Li, Honglei Wang, Yirui Guo, Xiaotong Wang, Hongmei Wu, Qing Zhang, Li Liu, Ge Meng, Shunming Zhang, Shaomei Sun, Ming Zhou, Qiyu Jia, Kun Song, Anna Stubbendorff, Yeqing Gu, Kaijun Niu

**Affiliations:** 1 Tianjin First Center Hospital Health Department, Tianjin, China; 2 School of Public Health of Tianjin, University of Traditional Chinese Medicine, 10 Poyanghu Road, West Area, Tuanbo New Town, Jinghai District, Tianjin, 301617 China; 3 School of Integrative Medicine, Tianjin University of Traditional Chinese Medicine, Tianjin, China; 4 Nutritional Epidemiology Institute, School of Public Health, Tianjin Medical University, Tianjin, China; 5 Wuqing District Centers for Disease Control and Prevention, Tianjin, China; 6 Health Management Centre, Tianjin Medical University General Hospital, Tianjin, China; 7 Nutritional Epidemiology, Department of Clinical Sciences Malmö, Lund University, Malmö, Sweden; 8 Institute of Radiation Medicine, Chinese Academy of Medical Sciences & Peking Union Medical College, Tianjin, China

**Keywords:** Immunoglobulin concentrations, T2DM, Adults, Prospective study

## Abstract

Type 2 diabetes mellitus (T2DM) is a metabolic disorder characterized by hyperglycemia resulting from defects in insulin secretion and/or insulin action. Increasing evidence suggests that inflammation played an important role in the pathogenesis of T2DM. Prospective studies on the link between immunoglobulins concentrations and the risk of T2DM in adults are limited. We developed a cohort study including 7,093 adults without T2DM history. The incidence of T2DM was 16.45 per 1,000 person-years. Compared with the lowest quartiles, the hazard ratios (95% confidence intervals) of T2DM for the highest quartiles of IgG, IgE, IgM and IgA were 0.64 (0.48–0.85), 0.94 (0.72–1.23), 0.68 (0.50–0.92) and 1.62 (1.24–2.11) (*P* for trend was < 0.01, 0.84, 0.02 and < 0.0001), respectively, suggesting that serum IgG and IgM concentrations were inversely associated with the incidence of T2DM, and IgA levels were positively associated with the risk of T2DM in a general adult population.

## Introduction

Type 2 diabetes mellitus(T2DM), a kind of diabetes due to a progressive loss of adequate β-cell insulin secretion frequently on the background of insulin resistance [1].In recent times, there has been a dramatic increase in Type 2 Diabetes worldwide due to changes in lifestyle, urbanization, and the hastened aging process [2].The number of adults worldwide with diabetes was estimated to be one in eleven (425 million) by the most recent International Diabetes Federation diabetes atlas, and that number was predicted to increase to 629 million by 2045 [3].Furthermore, individuals with type 2 diabetes (T2DM) may experience chronic consequences such as retinopathy, nephropathy, and cardiovascular diseases (CVD) owing to the low-grade systemic inflammation [4, 5].Conversely, T2DM was linked to a higher risk of death from all causes [6]. Consideration of the high prevalence and severity of T2DM, the prevention of T2DM is imperative.

An increasing amount of research indicated that inflammation was a major factor in the etiology of type 2 diabetes [7].An essential insulin signaling pathway intermediary called Akt was inactivated as a result of the stimulation of the C-Jun N-terminal kinase (JNK) signaling pathway by tumor necrosis factor (TNF-) and interleukin 1 (IL-1) [8].TNF- may also activate nuclear factor kappa B (NF-B), a transcription factor that stimulates the production of numerous inflammatory cytokines that can result in insulin resistance, by activating the IB kinase (IKK) pathway [9].

A critical function of the plasma cells in the immune response is the production of immunoglobulins. Serum immunoglobulin concentrations are routinely measured in clinical settings because they offer crucial insights on humoral immune response [10]. A previous animal study has shown B cells to produce pathogenic immunoglobulin G(IgG) antibodies with subsequent induction of macrophage oxidative bursts, cytotoxicity and pro-inflammatory cytokine production. It has been demonstrated that these elements raise insulin resistance, which changes glucose metabolism [11].Epidemiological studies have confirmed the association between inflammatory biomarkers and the development of type 2 diabetes mellitus and its complications [12], and we know that one of the effects of immunoglobulin molecules is to activate phagocytosis in macrophages by stimulating the cell lysis process, and that pro-inflammatory cytokines in inflammatory markers are essential for these immune homeostatic processes, and that adipose tissue is a major site for the production of inflammatory biomarkers [12]. Furthermore, it has been shown that immunoglobulins stimulate mast cells, which also produce pro-inflammatory mediators [13]. Thus, obese adipose tissue produces inflammatory markers in which pro-inflammatory cytokines cause an increased infiltration of macrophages and immune cells that contribute to local and systemic chronic low-grade inflammation, which in turn causes pre-diabetic symptoms such as dysglycemia, dyslipidemia, insulin resistance, atherosclerotic infiltration, and progressive vascular endothelial damage [14–17], and which further contribute to the chronic low-grade inflammation, which leads to a loss of homeostatic regulation of the immune system perpetuating chronic inflammation and ultimately contributing to the development of T2DM and long-term complications of diabetes. Therefore, it is assumed that immunoglobulins may be involved in the pathogenesis of Type 2 Diabetes. The prevalence of Type 2 Diabetes was found to be correlated with higher levels of immunoglobulin A (IgA) and lower levels of immunoglobulin M (IgM) and IgG in a cross-sectional study [18].However, since exposure and disease are only evaluated at a single time point, it is hard to identify any causality between the two in any cross-sectional study [19].The study aimed to explore whether immunoglobulin levels in a general population were associated with the risk of developing Type 2 Diabetes, hence suggesting a basis for any potential disease prevention.

## Methods

### Participants

Tianjin Chronic Low-Grade Systemic Inflammation and Health (TCLSIH) Cohort Study began in 2007 and focused on the association between chronic low-grade systemic inflammation and the health status of a population living in Tianjin, China. The details of TCLSIH Cohort Study have been described previously [20]. Study protocols and procedures were approved by the medical committee of the Institutional Review Board of Tianjin Medical University (number: TMUhMEC201430). All participants had provided written informed consent before participation in the study.

**Fig. 1:**
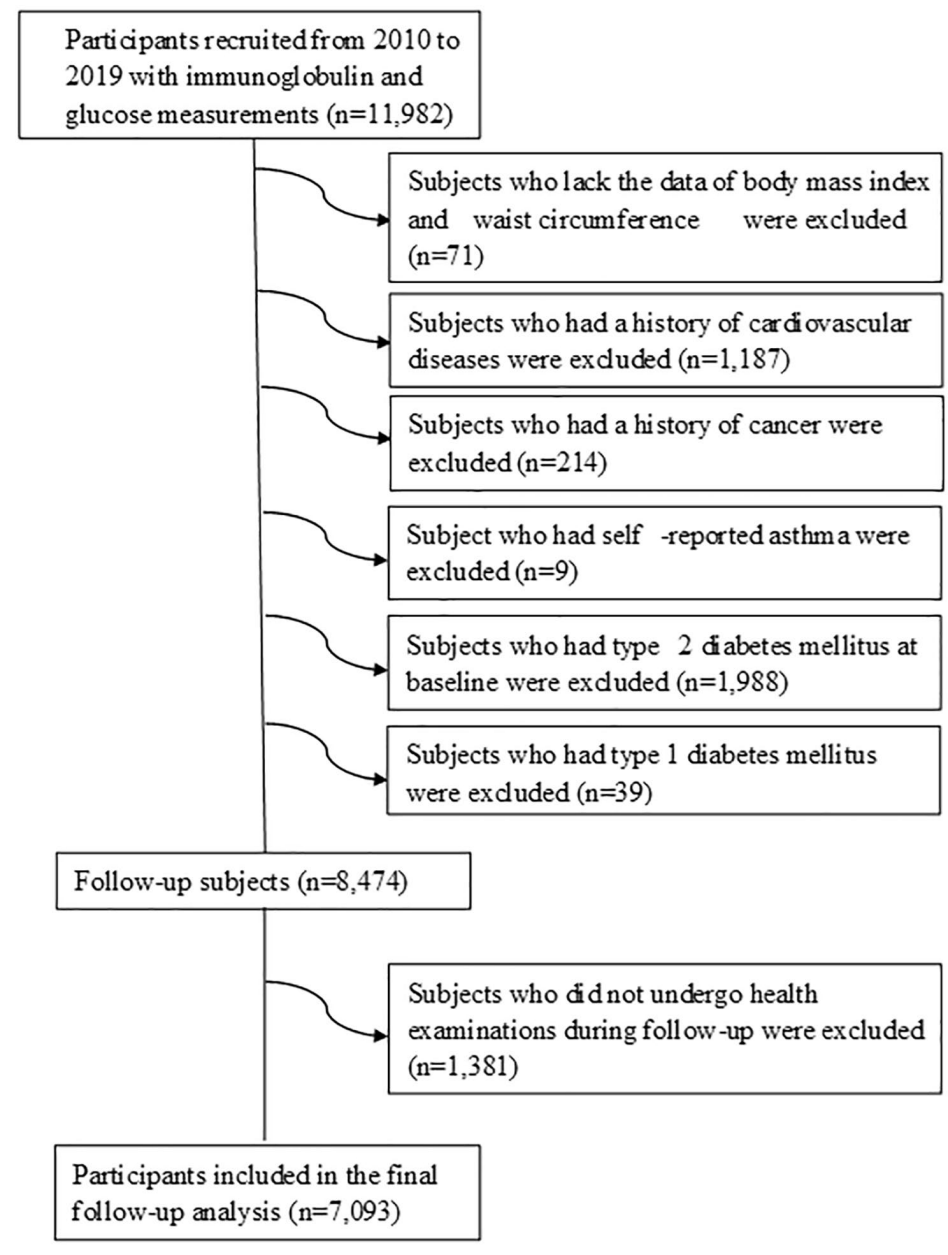
Selection of the study population, Tianjin Chronic Low-grade Systemic Inflammation and Health (TCLSIHealth) cohort study, 2010–2019

Data from the TCLSIH Cohort Study from 2010 to 2019 was used in the present study. The process for selecting participants was shown in Fig. 1. A total number of 11,982 participants received at least one physical examination. For the follow-up analysis, we excluded individuals who lack the data on body mass index (BMI), waist circumference (WC) (*n* = 71), or who had a history of CVD (*n* = 1,187) or cancer (*n* = 214) or self-reported asthma (*n* = 9). Moreover, subjects were excluded at baseline if they already had T2DM (*n* = 1,988), or type 1 diabetes mellitus (*n* = 39) or did not undergo health examinations during follow-up (*n* = 1,381). After these exclusions, the final cohort study comprised 7,093 participants (follow-up rate 83.7%, follow-up range: 1–9 years, mean duration of follow-up 4.52 years).

### Assessment of serum immunoglobulins

Serum immunoglobulins concentrations were determined by the immunonephelometric technique using IMMAGE 800 immunochemistry system. The detection limits of the analyzer for IgG, (IgE), IgM and IgA were: 33.3–21,600 mg/dL, 5–30,000 IU/mL, 4.2–14,400 mg/dL and 6.7–25,200 mg/dL, respectively. The manufacturer’s reference intervals for healthy adults were presented as follows: IgG 751–1560 mg/dL, IgE < 165 IU/mL, IgM 46–304 mg/dL and IgA 82–453 mg/dL. Serum immunoglobulin levels were assessed yearly during the follow-up.

### **Assessment of T2DM**

During the yearly follow-up, fasting plasma glucose (FPG) levels were measured with a glucose oxidase method. Two-hour plasma glucose was measured during an oral glucose tolerance test (OGTT). Glycosylated hemoglobin (HbA1c) was determined using a chromatography analyzer (HLC-723 GB; Tosoh, Tokyo, Japan). T2DM was classified as FPG level 7.0 mmol/L (126 mg/dL) and/or 2-h PG value in the OGTT 11.1 mmol/L (200 mg/dL) and/or HbA1c 6.5% or a history of T2DM, per the American Diabetes Association guidelines [21].

### Assessment of other variables

Total cholesterol (TC) and triglycerides (TG) were measured enzymatically. Low-density lipoprotein cholesterol was assayed using the polyvinyl sulfuric acid precipitation method and high-density lipoprotein cholesterol was determined by the chemical precipitation method using a Cobas 8000 modular analyzer (Roche, Mannheim, Germany). Serum alanine aminotransferase (ALT) was measured by the International Federation of Clinical Chemistry method. The estimated glomerular filtration rate (eGFR) is calculated by the modified Modification of Diet in Renal Disease (MDRD) equation for Chinese patients with chronic kidney disease (30): eGFR (ml/min per 1.73 m^2^) = 170 × Scr^− 1.234^ (mg/dl) × age^− 0.179^ × 0.79 (if female). Serum high-sensitivity C-reactive protein (Hs-CRP) levels were measured by the immunonephelometric assay using the Roche/Hitachi 917 analyzer (Roche, Mannheim, Germany), and expressed as mg/L. The eosinophil proportion was determined on a hematology analyzer XE-2100 (Sysmex, Kobe, Japan). Fibrinogen (FIB) was measured by using the STA-R evolution coagulation analyzer. The erythrocyte sedimentation rate (ESR) was measured by the Westergren method. Blood pressure was measured in the upper right arm using an automatic electronic sphygmomanometer (TM-2655P; A&D Company, Ltd., Tokyo, Japan), after a rest of 5 min in a seated position. The mean of the two measurements was calculated. Anthropometric variables (body height, weight and WC) were measured in standard ways. Weight and height were used to calculate BMI (kg/m^2^).

Lifestyle factors including smoking and drinking habits, family history of diseases, as well as self-reported inflammatory diseases (gastritis, chronic cholecystitis, nephritis, rheumatoid arthritis, gout, etc.), were obtained from a health-related questionnaire. Three categories were used to categorize people’s smoking status: “smoker,” “ex-smoker,” and “non-smoker.” Drinking status was classified as “everyday”, “sometimes”, “ex-drinker” or “non-drinker”.

### Statistical analysis

The distribution of continuous variables was non-normal. The natural log transformation was used to transform skewed data to approximately conform to normality. Descriptive data are presented as the geometric mean (95% confidence intervals, CI) for continuous variables and as a percentage for categorical variables. Differences between participants with and without T2DM were examined using analysis of covariance for continuous variables and logistic regression analysis for proportional variables. The incidence of T2DM was used as a dependent variable, and the quartiles of immunoglobulin were used as independent variables. Cox proportional hazards model was used to assess hazard ratios (HRs) and 95% CIs of T2DM for immunoglobulin levels (IgG, IgE, IgM and IgA). Model 1 was a crude model. Model 2 was adjusted for age, sex and BMI. Model 3 was further adjusted for WC, smoking status, drinking status, metabolic syndrome, eosinophil proportion counts, inflammatory diseases, family history of CVD, hypertension, hyperlipidemia, T2DM and immunoglobulin mutually adjusted. Model 4 was adjusted for variables in model 3 plus high-sensitivity C-reactive protein; estimated glomerular filtration rate; fibrinogen; erythrocyte sedimentation rate and alanine aminotransferase. Model 5 was adjusted for variables in model 4 plus fast blood glucose. The statistical analyses were conducted with SAS 9.3 version for Windows (SAS Institute Inc., Cary, NC, USA). Two-tailed *P* < 0.05 was considered statistically significant.

## Results

At the 9-year follow-up, 458 participants developed T2DM during the follow-up. The median duration of follow-up was 3.00 years. The incidence of T2DM was 16.45 per 1,000 person-years.

Table 1 shows age- and sex-adjusted characteristics of participants by T2DM status. Compared with participants without T2DM, those with T2DM are more likely to be male, tended to be older, to have higher levels of BMI, WC, SBP, DBP, TG, Alt, eGFR, Hs-CRP, FBG, PBG2h, HbA1c, WBC and IgA (*P* for trend < 0.01), but HDL, IgG, IgM and triglyceride-glycemic index (TyG index) were lower (*P* for trend < 0.001). The proportions of metabolic syndrome, hypertension, hyperlipidemia, nonalcoholic fatty liver disease, inflammatory diseases, ex-drinker, family history of CVD, hypertension and T2DM were higher in T2DM group. Apart from these results, no significant difference was observed between the two groups.

**Table 1:** Age- and sex- adjusted participant characteristics by T2DM status (*n* = 7,093) a

	T2DM status		*P* value ^b^
	No	Yes	
No. of subjects	6,635	458	-
Age (y)	48.5 (48.2, 48.7) ^c^	53.4 (52.4, 54.5)	< 0.0001
Sex (males, %)	58.9	77.1	< 0.0001
BMI (kg/m^2^)	25.0 (24.9, 25.1)	26.8 (26.5, 27.1)	< 0.0001
WC (cm)	83.7 (83.5, 83.9)	86.8 (86.0, 87.6)	< 0.0001
SBP (mmHg)	119.3 (118.9, 119.7)	122.6 (121.2, 124.1)	< 0.0001
DBP (mmHg)	76.4 (76.2, 76.7)	78.5 (77.5, 79.5)	< 0.001
TC (mmol/L)	4.90 (4.88, 4.93)	4.97 (4.88, 5.05)	0.19
TG (mmol/L)	1.27 (1.25, 1.28)	1.60 (1.53, 1.69)	< 0.0001
TyG index	8.52(8.14, 8.91)	8.96(8.65, 9.34)	< 0.001
LDL (mmol/L)	2.94 (2.92, 2.96)	2.96 (2.88, 3.04)	0.57
HDL (mmol/L)	1.36 (1.35, 1.36)	1.27 (1.24, 1.30)	< 0.0001
ALT (U/L)	19.8 (19.6, 20.1)	23.2 (22.2, 24.2)	< 0.0001
eGFR (ml/min per 1.73m^2^)	105.6 (105.2, 106.1)	108.3 (106.6, 110.2)	< 0.01
Hs-CRP (mg/L)	0.82 (0.80, 0.84)	1.10 (1.00, 1.20)	< 0.0001
FBG (mmol/L)	4.96 (4.95, 4.97)	5.62 (5.57, 5.67)	< 0.0001
PBG2h (mmol/L)	5.97 (5.95, 6.00)	7.31 (7.19, 7.43)	< 0.0001
HbA1c (%)	5.43 (5.43, 5.44)	5.84 (5.82, 5.88)	< 0.0001
WBC (×1000 cells/mm^3^)	5.40 (5.36, 5.43)	5.60 (5.50, 5.70)	< 0.01
FIB (g/L)	2.68 (2.67, 2.69)	2.72 (2.68, 2.77)	0.08
ESR (mm/h)	6.80 (6.70, 7.00)	6.40 (6.00, 6.90)	0.11
Eosinophil proportion counts (%)	1.79 (1.75, 1.83)	1.80 (1.60, 1.90)	0.74
IgG (mg/dL)	1199.8 (1194, 1205.5)	1156.7 (1135.8, 1178)	< 0.001
IgE (IU/mL)	30.8 (29.7, 31.8)	31.7 (28.0, 36.0)	0.63
IgM (mg/dL)	97.8 (96.7, 98.9)	88.8 (85.1, 92.6)	< 0.0001
IgA (mg/dL)	216.1 (213.9, 218.4)	231.9 (223.1, 241.2)	< 0.001
Metabolic syndrome (%)	26.5	62.6	< 0.0001
Hypertension (%)	30.2	50.4	< 0.0001
Hyperlipidemia (%)	59.3	72.7	< 0.001
NAFLD (%)	38.7	72.7	< 0.0001
Inflammation disease (%)	22.6	29.4	< 0.01
Smoking status (%)			
Smoker	27.2	36	0.57
Ex-smoker	4.92	8.88	0.12
Non-smoker	67.9	55.1	0.15
Drinker (%)			
Everyday	4	6.02	0.53
Sometime	53.9	58.1	0.9
Ex-drinker	3.43	6.02	< 0.01
Non-drinker	38.6	29.9	0.16
Family history of diseases (%)			
CVD	36.8	46.5	< 0.001
Hypertension	52.9	60.5	< 0.01
Hyperlipidemia	0.45	0	0.98
T2DM	21.9	37.1	< 0.0001

^a^ T2DM, type 2 diabetes mellitus; BMI body mass index; WC waist circumference; SBP, systolic blood pressure; DBP, diastolic blood pressure; TC, total cholesterol; TG, triglycerides; TyG index, triglyceride-glucose index; LDL, low density lipoprotein cholesterol; HDL, high-density lipoprotein-cholesterol; ALT, alanine aminotransferase; eGFR, estimated glomerular filtration rate; hs-CRP, high-sensitivity C-reactive protein; FBG, fast blood glucose; PBG2h, 2 h postprandial blood glucose; HbA1c, glycated hemoglobin; WBC, white blood cell count; FIB, fibrinogen; ESR, erythrocyte sedimentation rate; NAFLD, nonalcoholic fatty liver disease; IgG, immunoglobulin G; IgE, immunoglobulin E; IgM, immunoglobulin M; IgA, immunoglobulin A; CVD, cardiovascular disease.

^b^ Analysis of covariance or logistic regression analysis.

^c^ Geometric mean (95% confidence interval) (all such values).

The crude and adjusted association between immunoglobulins concentration and the incidence of T2DM are presented in Table 2. In model 1, the crude model, the risk of diabetes mellitus gradually decreased with the graded increase in IgG concentration (*P* for trend < 0.001), and this trend obtained after correcting for a number of variables in models 2, 3, and 4, respectively, was still significant (*P* for trend < 0.01), and we finally, in the fully adjusted model (model 5), similar results were obtained - the HRs (95% CIs) for the elevated quartiles were 1.00, 0.89 (0.70, 1.13), 0.77 (0.59, 1.01), and 0.64 (0.48, 0.85), respectively (*P* for trend < 0.01); in contrast, the results of fully adjusted modeling for IgE showed no significant difference in T2DM incidence between patients with different grades of IgE concentration (*P* for trend < 0.01). incidence were not significantly different from each other (*P* for trend = 0.84) and did not reflect the association well even in the crude model (*P* for trend = 0.05); in addition, the results of all five models showed a prospective association between IgM and T2DM (*P* for trend < 0.05), and model 5 corrected for a range of covariates, and the results still showed that higher concentrations of IgM predicted a lower risk of T2DM incidence The HRs (95% CIs) for the elevated quartiles of this model were 1.00, 0.89 (0.71, 1.13), 0.88 (0.68, 1.14), and 0.68 (0.50, 0.92), respectively (*P* for trend value = 0.02); in particular, the crude model showed a positive association between IgA concentration and the risk of T2DM incidence, which was further analyzed by proportional risk regression adjusting for a series of confounders further analyzed the prospective relationship between IgA and later development of T2DM, which was supported by the HRs (95% CIs) of the fully adjusted modeled outcome data-elevated quartiles of 1.00, 0.93 (0.70, 1.25), 1.33 (1.01, 1.75), and 1.62 (1.24, 2.11) (*P* for trend < 0.001), respectively) that was established.

**Table 2:** Adjusted associations of quartiles of serum immunoglobulins concentrations to T2DM (*n* = 7,093) a

	Quartiles of immunoglobulins concentrations				*P* for trend ^b^
	Level 1	Level 2	Level 3	level 4	
IgG concentration (mg/dL, range)	517.0–1,050.0	1,060.0–1,200.0	1,210.0–1,340.0	1,350.0–3,330.0	-
No. of subjects	1,868	1,762	1,607	1,856	-
Person-years of follow-up (n)	7,492	6,762	6,538	7,054	-
No. of T2DM	163	118	91	86	-
Model 1 ^c^	1.00	0.80 (0.63, 1.01) ^d^	0.64 (0.50, 0.83)	0.56 (0.43, 0.72)	< 0.0001
Model 2 ^e^	1.00	0.88 (0.70, 1.12)	0.74 (0.57, 0.96)	0.66 (0.51, 0.86)	< 0.001
Model 3 ^f^	1.00	0.88 (0.69, 1.12)	0.72 (0.55, 0.94)	0.63 (0.48, 0.84)	< 0.001
Model 4 ^g^	1.00	0.89 (0.70, 1.13)	0.73 (0.56, 0.95)	0.62 (0.47, 0.83)	< 0.01
Model 5 ^h^	1.00	0.89 (0.70, 1.13)	0.77 (0.59, 1.01)	0.64 (0.48, 0.85)	< 0.01
IgE concentration (IU/mL, range)	5.00-10.3	10.4–28.2	28.3–80.4	80.5-4,420.0	-
No. of subjects	1,777	1,772	1,770	1,774	-
Person-years of follow-up (n)	7,257	6,862	6,915	6,812	-
No. of T2DM	108	109	111	130	-
Model 1 ^c^	1.00	1.06 (0.81, 1.38)	1.08 (0.83, 1.40)	1.27 (0.99, 1.64)	0.05
Model 2 ^e^	1.00	1.00 (0.77, 1.31)	0.93 (0.71, 1.22)	1.05 (0.81, 1.36)	0.56
Model 3 ^f^	1.00	0.99 (0.75, 1.29)	0.94 (0.72, 1.24)	1.06 (0.81, 1.37)	0.48
Model 4 ^g^	1.00	1.00 (0.77, 1.31)	0.93 (0.71, 1.21)	1.04 (0.80,1.36)	0.59
Model 5 ^h^	1.00	0.97 (0.74, 1.28)	0.88 (0.67, 1.16)	0.94 (0.72, 1.23)	0.84
IgM concentration (mg/dL, range)	7.18–67.8	67.9–93.8	93.9–129.0	130.0-583.0	-
No. of subjects	1,774	1,779	1,744	1,796	-
Person-years of follow-up (n)	6,908	6,948	6,805	7,185	-
No. of T2DM	161	132	102	63	-
Model 1 ^c^	1.00	0.82 (0.65, 1.03)	0.64 (0.50, 0.82)	0.38 (0.28, 0.51)	< 0.0001
Model 2 ^e^	1.00	0.92 (0.73, 1.16)	0.87 (0.67, 1.12)	0.64 (0.47, 0.87)	< 0.01
Model 3 ^f^	1.00	0.92 (0.73, 1.16)	0.90 (0.70, 1.16)	0.70 (0.52, 0.95)	0.02
Model 4 ^g^	1.00	0.90 (0.71, 1.13)	0.90 (0.69, 1.16)	0.70(0.52, 0.95)	0.03
Model 5 ^h^	1.00	0.89 (0.71, 1.13)	0.88 (0.68, 1.14)	0.68 (0.50, 0.92)	0.02
IgA concentration (mg/dL, range)	6.67–166.0	167.0-222.0	223.0-289.0	290.0–1,230.0	-
No. of subjects	1,789	1,770	1,735	1,799	-
Person-years of follow-up (n)	7,019	7,114	6,910	6,802	-
No. of T2DM	102	88	112	156	-
Model 1^c^	1.00	0.84 (0.63, 1.12)	1.16 (0.88, 1.52)	1.49 (1.16, 1.93)	< 0.0001
Model 2 ^e^	1.00	0.86 (0.64, 1.14)	1.14 (0.87, 1.49)	1.44 (1.13, 1.86)	< 0.001
Model 3 ^f^	1.00	0.93 (0.70, 1.24)	1.29 (0.98, 1.70)	1.71 (1.31, 2.22)	< 0.0001
Model 4 ^g^	1.00	0.93 (0.70, 1.24)	1.30 (0.99, 1.72)	1.70 (1.31, 2.22)	< 0.0001
Model 5 ^h^	1.00	0.93 (0.70, 1.25)	1.33 (1.01, 1.75)	1.62 (1.24, 2.11)	< 0.001

^a^ T2DM, type 2 diabetes mellitus; IgG, immunoglobulin G; IgE, immunoglobulin E; IgM, immunoglobulin M; IgA, immunoglobulin A.

^b^ Analysis by Cox proportional hazards model.

^c^ Model 1 was crude model.

^d^ Hazard ratio (95% confidence interval) (all such values).

^e^ Model 2 was adjusted for age, sex and obesity.

^f^ Model 3 was adjusted for variables in model 2 plus waist circumference, smoking status, drinking status, hypertension, hyperlipidemia, nonalcoholic fatty liver disease, eosinophil proportion counts, inflammatory diseases, family history of cardiovascular disease, hypertension, hyperlipidemia and T2DM and immunoglobulin mutually adjusted.

^g^ Model 4 was adjusted for variables in model 3 plus high-sensitivity C-reactive protein, estimated glomerular filtration rate, fibrinogen, erythrocyte sedimentation rate and alanine aminotransferase.

Model 5 was adjusted for variables in model 4 plus fast blood glucose.

In addition, we performed a sensitivity analysis excluding participants with inflammatory diseases (*n* = 1,490) in the final model. The results were similar to our previous analysis (data not shown).

## Discussion

To the best of our knowledge, our study is the first prospective cohort study investigating the association between immunoglobulin concentrations and Type 2 Diabetes incidence in a population-based cohort. With relevant confounders adjusted for, we found lower IgG and IgM levels and higher IgA levels to be significantly associated with increased risk of Type 2 Diabetes, while this was not observed for IgE concentrations.

IgG is the predominant immunoglobulin in the body and provides the majority of antibody-based immunity against invading pathogens. A cross-sectional study conducted in Italy showed a correlation between blood IgG2 concentrations and insulin-stimulated glucose disposal [22]. Additionally, a previous study showed that in T2DM patients, lower blood IgG levels were predictive of non-diabetic renal disease [23]. Serum IgG levels were observed to be negatively correlated with the risk of T2DM in the current investigation [24], which aided in the development of T2DM, this is consistent with our findings. IgE often participates in hypersensitivity and allergic reactions, binding to specific Fc receptors. Several lines of evidence demonstrated that IgE may play a role in inflammatory disease. IgE was a significant risk factor for T2DM after adjusting for age, sex, hypertension, BMI, cholesterol, high-sensitivity C-reactive protein, and mast cell chymase and tryptase [25]. IgE was linked to metabolic syndrome plus T2DM, according to a small cross-sectional study [OR: 2.38 (95% CI, 1.13–5.02)] [26]. However, in our cohort study with 7,093 participants, no significant difference was observed between IgE concentration and the incidence of T2DM. Reasons for the discrepancy between these findings remain unclear, it may be because the sample size of the study was only 340, which is small compared to our 7093 cases, and the small sample size resulted in less efficient testing and therefore less sensitivity to synergistic effects when analyzing the interactions of the various factors, and because our cohort study was rigorously designed and prospective compared to the cross-sectional study. Moreover, we considered and adjusted for confounders more thoroughly and also included inflammatory markers such as high-sensitivity C-reactive protein, estimated glomerular filtration rate, fibrinogen, erythrocyte sedimentation rate and alanine aminotransferase et al.

In B Cell development and in the primary antibody response, IgM is the first and largest class of immunoglobulins expressed [27]. IgM levels were found to decrease as blood glucose levels rose in a prior study [28]. Additionally, a cross-sectional investigation with 147 obese participants and 111 age- and sex-matched controls of normal weight revealed a substantial drop in IgM levels in the obese group [29]. Most of the IgM in plasma is produced by the B-1B subgroup of B cells [27]. A recent animal investigation found an inverse relationship between insulin resistance and plasma IgM levels [30]. And CD20 + CD27 + CD43 + CD70 − B-cell subset in umbilical cord and peripheral blood that is characterized by spontaneous secretion of IgM antibodies. Alternatively, daratumumab and elotuzumab allow specific targeting of antibody-secreting cells, while largely preserving B-cell populations [31]. Additionally, in Type 2 Diabetes patients, a study has shown circulating B cells (mostly being of B-2 subset of B cells) to be skewed toward a pro-inflammatory phenotype after Toll-like receptor (TLR) stimulation.

According to the authors of the aforementioned study, this change may result in a decrease in the number of B-1B subsets and, in turn, in IgM secretion. Finally, the same study proposed that variations in the up- and down-shift of anti-inflammatory cytokines may result in enhanced inflammation due to a shift in TLR function in B cells [32]. Relatively, a review summarizes the evidence for infiltration of adipose tissue by cells of the adaptive immune system in the context of obesity [15]. The most prevalent antibody in humans is IgA [33].In line with the findings of the cross-sectional investigation [18], we discovered that a greater IgA concentration was linked to an increased risk of type 2 diabetes.

The information on insulin resistance was not measured in the present study, which was important to explain the mechanism. Since they do not have insulinemia, we cannot calculate the HOMA index. However, we calculated the TyG index instead and found that it was significantly higher in diabetic group than in non-diabetic group. A cross-sectional study from India in diabetic patients with different groups of glycemic control showed that the value of TyG index was higher in the poorly controlled group [34], which corroborates our results to some extent. Elevated triacylglycerols in diabetic patients contribute to poor glycemic control by affecting glucose metabolism [35], and the TyG index not only reflects glycemic control but is also a good predictor of insulin resistance [34]. Babic N et al. have also shown that the TyG index can be used to assess the degree of insulin resistance in T2DM [36], and similarly, more recent studies have suggested that it may be a useful tool for identifying people at high risk of IR and future diabetes mellitus. Similarly, recent studies have suggested that the TyG index may be a useful tool for identifying people at risk for IR and future diabetes [37].C-reactive protein (CRP), a sensitive marker of systemic inflammation, has been shown to be increased in patients with type 2 diabetes mellitus [38], and it also predicts the development of diabetes [39]. An earlier cohort study had found an association between elevated fibrinogen, sialic acid, and stomatitis in subjects and a later diagnosis of diabetes [40], and more recently a 10-year longitudinal study from a Korean community reported that high leukocyte counts were predictive of type 2 diabetes [41]. A large cohort study in China illustrated that baseline serum albumin levels appear to be negatively associated with the risk of T2DM [42]. Thus, the inflammatory markers mentioned above can, in a sense, be combined to predict the occurrence of type 2 diabetes events. In addition, numerous studies have found an association between these markers and obesity or BMI [43–45].The current investigation represents the first cohort study to thoroughly examine the relationships between immunoglobulin concentrations and the risk of type 2 diabetes in a sizable adult population. However, our study had a limitation. Although we adjusted for as many as possible potential confounding factors, we cannot fully exclude the possibility of unmeasured or residual confounding.

## Conclusion

Serum IgG and IgM concentrations were inversely associated with the incidence of T2DM, and IgA levels were positively associated with the risk of T2DM in a general adult population. However, no significant association was observed between IgE concentration and the incidence of T2DM.

## Data Availability

No datasets were generated or analysed during the current study.

## Declarations

### Data sharing

Data described in the manuscript can be made available upon request pending application and approval by the chair of the steering committee for the cohort.
